# Analyzing the Sample Geometry Effect on Mechanical Performance of Drilled GFRP Connections

**DOI:** 10.3390/ma15082901

**Published:** 2022-04-15

**Authors:** Yongcheng Zhu, Hua Zhu, Viktor Gribniak

**Affiliations:** 1 Department of Steel and Composite Structures, Vilnius Gediminas Technical University (VILNIUS TECH), Sauletekio Av. 11, LT-10223 Vilnius, Lithuania; yongcheng.zhu@stud.vilniustech.lt; 2 Yancheng Institute of Technology, 1st Xiwangdadao Road, Yancheng 224051, China; yczh716@ycit.cn

**Keywords:** fiber-reinforced polymer, glass fibers, pultrusion, bolted connection, tensile tests, failure, finite element analysis

## Abstract

A considerable effort to understand the bolted joints’ mechanical behavior in pultruded profiles has existed in the literature over the past decades. However, most investigations focused on the single-bolt connections, and only a few works considered single-lap joints. This paper investigates the mechanical performance of a single-lap connection of pultruded glass fiber-reinforced polymer (GFRP) plates owning to the experimental data deficit in the literature. Tensile tests of specimens with different geometries generate a database comprising 80 single-bolt joints. The shear-out failure was predominant for the considered GFRP pultruded plates, with the end length mainly affecting the load-bearing capacity. Hart-Smith’s theoretical model overestimated the ultimate resistance of all considered joints—the exceptionally low efficiency of the GFRP composite points out the necessity of additional means for strengthening the drilled connections. Additionally, finite element (FE) software Abaqus simulated the bolted joints; this study employs the user-defined subroutine experimentally verified in the literature. In the considered examples, the ultimate resistance prediction error decreased from 25.7% to 2.9% with increasing the plate thickness (from 4 mm to 8 mm) and width (from 25 mm to 35 mm), which proves the reasonable adequacy of the simplified FE model and makes it a valuable reference for further bolted joints’ development.

## 1. Introduction

**Figure 1:**
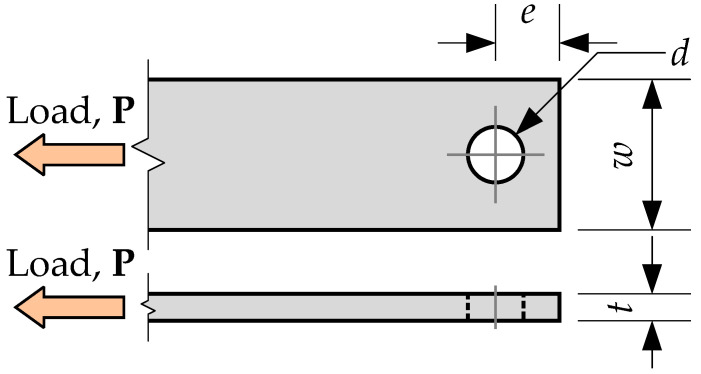
The connection geometry definition.

Fiber-reinforced polymer (FRP) composites become typical for lightweight structures due to the high strength-to-weight ratio, corrosion resistance, electromagnetic transparency, and ease of processing [1,2,3]. The FRP connections’ design guide [4] pointed out the adequacy of the linear model in approximating the mechanical behavior of FRP materials up to failure at the coupon level. However, coupon or structural shapes behave nonlinearly beyond certain load levels because of the differences in the joining methods and particular fiber and fabric layups. Thus, the mechanical models must account for the specific behavior of the FRP composite, and this understanding requires physical tests. Furthermore, although the FRP structural components have the shape of plates and profiles typical for steel elements, FRP material is highly vulnerable to the load orthogonal to the fiber orientation [5]. The review article [6] identified adapting the well-established design procedures for steel connections (based on years of experience with isotropic and homogeneous materials) to account for the heterogeneous and directional properties of FRPs as one of the most challenging problems. In addition, pultruded composites cannot redistribute loads through the yielding (characteristic for metals) and, hence, reduce the sensitivity to stress concentrations; the inherent brittle behavior of FRPs renders the fastening joints susceptible to premature damage. Reference [6] provides the following classification of the physical characteristics affecting the joints’ mechanical performance:

- *Geometric parameters* (Figure 1) include the width-to-diameter ratio, *w*/*d*, end distance-to-diameter ratio, *e*/*d*, and plate thickness, *t*;
- *Material parameters* group includes fiber and matrix type, fiber alignment, and laminate stacking sequence;
- *Joint configuration* describes shear panel number (single- or double-lap), number of bolts and bolt-rows, etc.;
- *Fastener parameters* include the fastener type and clearance of the hole;
- *Lateral restraint* describes bolt tightening and clamping area;
- *The design conditions* group specifies loading type, direction, duration, and failure mechanisms.

Hart-Smith [7] developed an analytical procedure for failure analysis of mechanically fastened composite bolted joints. With some modifications proposed by Rosner [8], this model describes the maximum stress, *σ*, in the joint shown in Figure 1 as follows:

(1) $$\sigma =k_{e}\cdot \frac{P}{t\cdot \left(w-d\right)}$$

where *k_e_* is the elastic stress concentration factor, which is described as follows [9]:

(2) $$k_{e}=\frac{w}{d}+\frac{d}{w}+0.5\cdot \left(1-\frac{d}{w}\right)\cdot \theta \approx \frac{w}{d}+\frac{d}{w}$$

with coefficient

(3) $$\theta =\left\{\begin{array}{c} \begin{array}{cc} \frac{w}{e}-1, & e/w\leq 1; \end{array}\\ \begin{array}{cc} 0, & e/w>1 \end{array}. \end{array}\right.$$

Notwithstanding the progress in the fastening technologies [6], the sample geometry effect on the efficiency of the bolted joints describes the continued discussion object. Remarkably, the efficiency description in metallic and FRP joints is also different—net strength describes the metallic connection performance, and the ultimate load-bearing capacity, **P***_u_*, describes the FRP connection effectiveness. In particular, the ratio of the joined (drilled) member capacity to the ultimate resistance of the undamaged element determines the joint efficiency parameter [4]:

(4) $$\eta =\frac{P_{u}}{w\cdot t\cdot f_{t}}$$

where *f_t_* is the tension strength of the FRP material.

The differences in the FRP manufacturing technologies and internal (reinforcement) structure cause everlasting discussions in the literature. İçten & Sayman [10] found that the *e*/*d* and *w*/*d* ratios have a similar effect on the bearing strength of the aluminum and glass fiber-reinforced polymer (GFRP) sandwich plates; the *w*/*d* ratio controls the failure mechanism, with the condition *e*/*d* ≥ 3 ensuring the full load-bearing performance. This observation supports results by Cooper & Turvey [11], which demonstrated that the shear damage of GFRP laminate occurred when *e*/*d* < 3, the hole extrusion occurred when *e*/*d* > 3, and the laminate shear damage replaced the hole extrusion at *e*/*d* > 4. Furthermore, the bolt clamping torque increased the failure load and reduced the critical end distance and plate width. Nhut et al. [12] investigated the bolt diameter effect on the load-bearing capacity of typical GFRP profiles manufactured in Japan; the damage mechanisms altered to the hole bearing failure at *w*/*d* > 5. At the same time, this study developed a reliable finite element (FE) model to predict the load-bearing capacity of the bolted connections with Hashin failure criteria and Lusas software. Similar profiles were also the object of research [13], which developed an efficient and reliable strengthening system for bolted connections, employing thin multiaxial glass fiber sheets. Eskenati et al. [14] experimentally demonstrated the bolted joints’ prominence regarding adhesive joints characterized by brittle failure; references [15,16,17] support this observation. The numerical model [14] with Abaqus software assumed the linear-elastic and transverse isotropy of GFRP material. The latter example is typical for the GFRP structural analysis—65% of studies reviewed in reference [18] employed the elastic material model to simulate such components.

References [19,20] applied elaborate probabilistic procedures to predict the ultimate resistance and simulation of random connection clearances in the bolted joints. Belardi et al. [21] developed a computationally efficient simulation tool representing stiffness components of the bolted region when a set of radially arranged customized beams describes the user-defined element. The first-order shear deformation plate theory [22] described the elastic contribution of carbon FRP laminate; the bolt stiffness model accounts for its shank, bolt head, and bolt hole bearing deformation. Liu et al. [23] introduced an improved 2D finite element model accounting for the secondary bending effect and adding holes in the model that improve the bolted joint stiffness prediction adequacy. The study [24] demonstrated that an additional adhesive (hybrid) connection could increase the load-bearing capacity of the single-bolt joint by 10%. The considered situations represented CFRP laminate, which failure is not sensitive to the fiber orientation in the polymer matrix because of the appropriately designed stacking sequence of the unidirectional layers of the laminated plates [25,26,27]. However, the mechanical performance prediction of the drilled connections of pultruded FRP components is problematic because of the material anisotropy [4]. Matharu & Mottram [28] found that the pin-bearing strength of the FRP specimens loaded in the direction of pultrusion could exceed two times the load-bearing capacity of the samples loaded in the orthogonal direction. The article [29] provides a valuable reference to the bolted joint database of pultruded FRP components consisting of more than 1000 tested cases.

From the industrial point of view, the well-developed pultrusion technologies enable fabricating a large volume of structural components at low operating costs, high production rate, high product reproducibility, and dimensional tolerances [30]. The pultrusion allows the distribution of a high volume of continuous mechanically resistant filaments in a polymer matrix that protects the reinforcement from mechanical and environmental impacts. Still, unfortunately, the application of FRP profiles is limited to simple structural cases [31]; for instance, there is no mature specification for the connection of composite materials in China.

Notwithstanding gathered datasets and comprehensive reviews of pultruded FRP composites fastening reported in the literature [6,29], a considerable effort still exists over the past decades to develop an understanding of the bolted joints’ mechanical behavior. For example, Turvey [32] found that most experimental investigations focused on the single-bolt joint performance; however, only a few works have been reported on single-lap joints. Thus, there are currently no quantitative data on the mechanical performance of such connections. Furthermore, the manufacturing technologies substantially vary the mechanical properties of FRP materials, complicating the practical engineering applications. Recognizing this situation motivates the present experimental study.

This paper investigates the mechanical performance of a single-bolt single-lap connection of pultruded GFRP plates produced in China, determining the safe range of the joint geometry. The research variables are the plate thickness and width, end distance, and bolt diameter. The test matrix extends the characteristic *e*/*d* and *w*/*d* ratios’ ranges assumed in the reference [32]. In the present study, the experimentally verified subroutine [33] for the laminated composite describes the pultruded GFRP failure assuming it is a single-layer plate and distinguishing fibers and polymer matrix damage processes. This FE simulation and empirical model of unidirectional FRP composite [7,8,9] describe the theoretical reference for estimating the physical connection efficiency.

## 2. Materials and Methods

### 2.1. Test Samples

The tests employ the GFRP composite plate manufactured by Henan Embrace Co. (Henan, China), comprising the pultruded core of unidirectional glass filaments covered with thin multiaxial glass fiber sheets. The fiber-reinforcement percentage provided by the manufacturer is 65%. Table 1 describes the experimentally assessed material properties of the GFRP composite.

**Figure 2:**
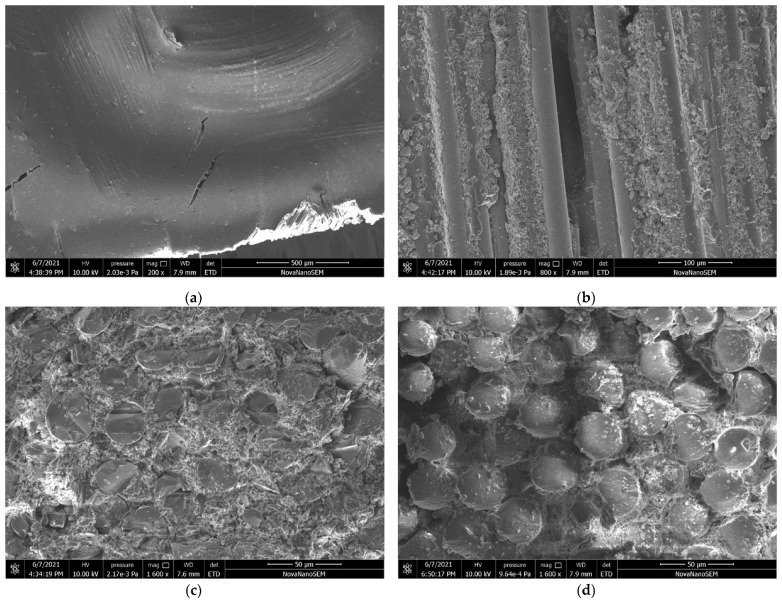
The SEM images of internal fiber structure: (**a**) The laminated surface (magnification 200×); (**b**) Longitudinal fiber view (magnification 800×); (**c**,**d**) Images normal to the pultrusion direction (magnification 1600×).

**Figure 3:**
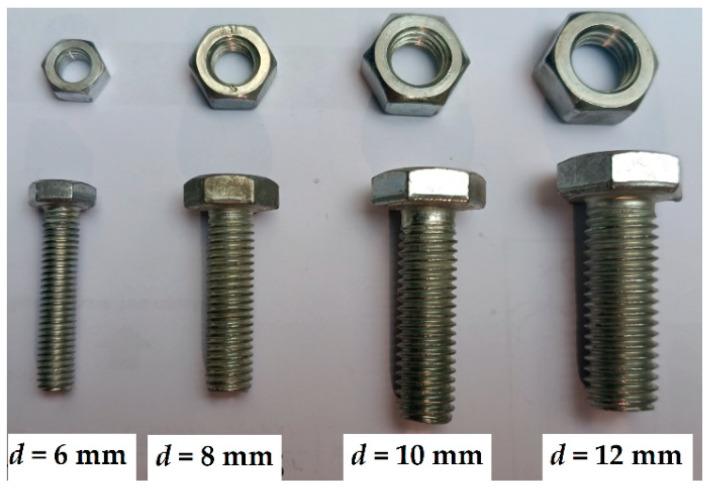
The connection bolts Grade 4.6 with nuts.

The test program includes four types of plate samples—Table 2 defines the geometric parameters of the different specimen types, and Figure 1 describes the notations. The test coupons were cut from the plates having the corresponding thickness. The sample appearance and filament structure were checked visually; the internal structure of several samples was verified using the scanning electron microscopy (SEM) technique. Figure 2 shows characteristic views of the specimen structure, demonstrating no apparent damage to the fibers. Each connection group (Table 2) consists of five nominally identical samples. The drilled hole diameter was the test variable. Four diameters (i.e., 6 mm, 8 mm, 10 mm, and 12 mm) are considered. The fasteners are the ordinary bolts Grade 4.6 (Chinese standard GB/T 152.4-1988) with a nominal strength of 400 MPa and yield strength of 240 MPa; a 5 Nm torque was applied to tighten the nuts. Figure 3 shows the bolt samples.

**Figure 4:**
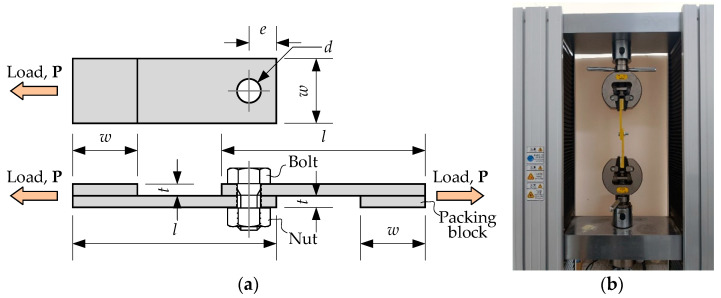
Tensile testing: (**a**) The loading scheme; (**b**) The test setup.

The holes were drilled with the alloy triangle piercer and polished with a diamond mill. This procedure was carried out carefully to avoid damage to the GFRP plate and minimize the bolt clearance. Table 3 describes the sample configuration. In this table, the specimen notation determines the specimen type (Table 2) and the diameter of the hole. Figure 4a shows the specimen configuration. As mentioned in Section 1, the test matrix extends the *e*/*d* and *w*/*d* ranges assumed in the literature except for the *e*/*d* < 2, which Turvey [32] found inefficient.

### 2.2. Test Method

The standard [34] described the testing procedure and loading conditions. An electro-mechanic 100 kN universal testing machine, Byes 2100 (Bangyi Precision Measuring Instruments, Shanghai, China), under the displacement control and 2 mm/min speed, loaded the specimens until the failure. The testing machine recorded the load and displacement every second. Adhesively connected packing blocks made from the same GFRP plate as the tested sample protected the loaded end in the testing grips and reduced the load eccentricity. Figure 4 shows the loading scheme and test setup, which correspond to the test rig from work [32].

## 3. Test Results

Table 4 summarizes the test results and describes the failure mode, estimated by following the classification [4,9]. The damage classification is based on most modes observed in five identical samples: “BF” stands for the bolt shear failure, “C” denotes the cleavage failure, “S” designates the shear-out failure, and “D” represents the delamination damage of the end zone.

In Table 4, **P***_u_* determines the maximum experimental load, and *σ_m_* is the corresponding mean stresses in the GFRP plate determined as follows:

(5) $$\sigma_{m}=\frac{P_{u}}{d\cdot t}$$

**Figure 5:**
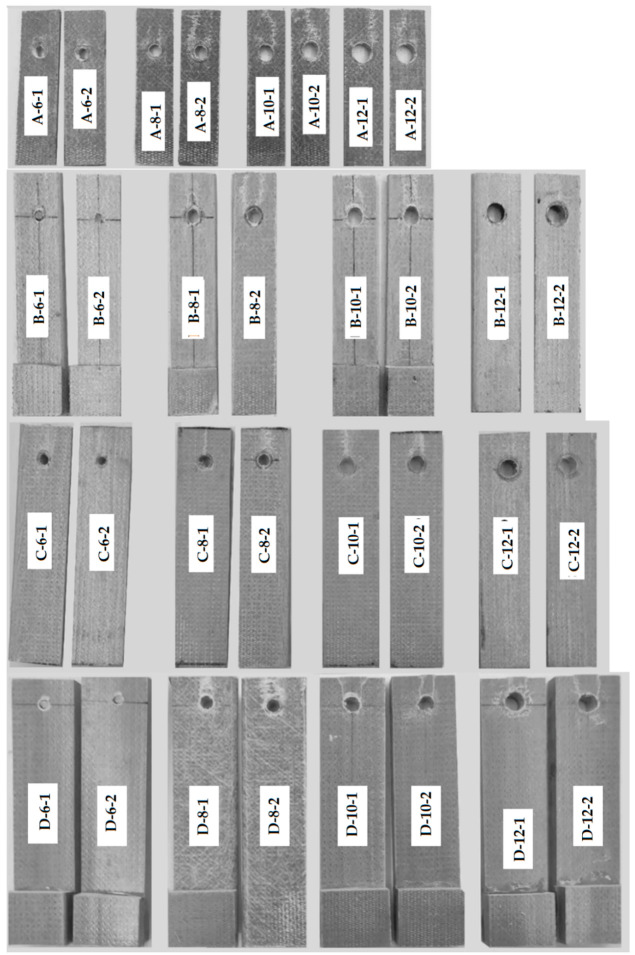
Failure patterns of the selected joints (scaled to represent actual size).

Figure 5 demonstrates the failure patterns of the selected joints. The A-6 samples show the bearing failure signs with damages arched around the holes. The cracks formed at the bolt support and extended to the laminate end are characteristic of the A-10 and A-12 joints, evidencing the cleavage laminate failure. At the same time, some joint samples (e.g., A-10-1) had cracks on both sides of the perforation, though only one crack path fully developed. The A-8 joints represent the transition situation between A-6 and A-10 cases; still, the cleavage failure has not been reached.

**Figure 6:**
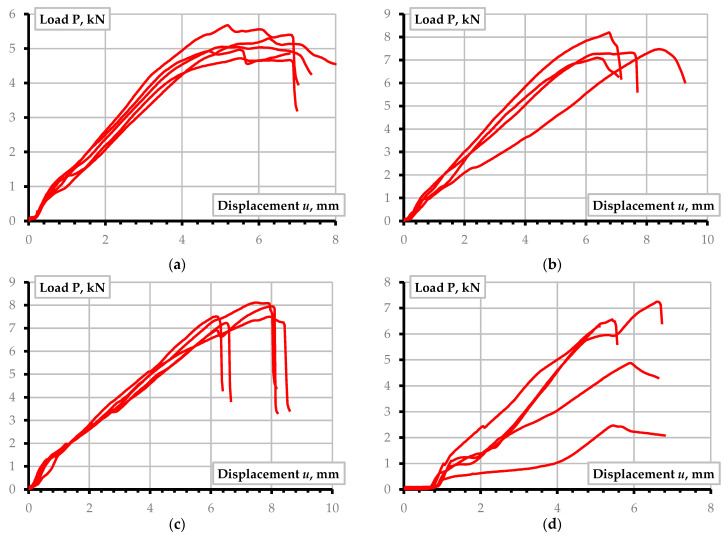
Characteristic load-displacement diagrams of the tested joints: (**a**) A-6 connection type; (**b**) A-8 type; (**c**) A-10 connections; (**d**) A-12 joints. Note: the diagrams of each graph show responses of five nominally identical specimens.

The bolt failure has resulted from the B-6 samples’ tests. Still, the B-8 and B-10 specimens demonstrate the shear-out failure signs. Notwithstanding the failure-confined effect of the external laminate layer, the GFRP fragments separated by the parallel cracks were extruded in the B-8-2 and B-10-2 samples. This failure is also typical for the C-Type connections—it appeared in more than half of the specimens comprising each group of the joint (Table 4). The relative reduction in the end distance, *e*, clarifies the failure mechanisms in the D-Type joints. The D-10 and D-12 specimens demonstrate parallel cracks development and shearing of the separated laminate fragment. The D-8 samples possess the transition from the bearing to the shear-out failure case. Figure 6 shows the characteristic load-displacement diagrams of the test specimens.

## 4. Discussion of the Results

**Figure 7:**
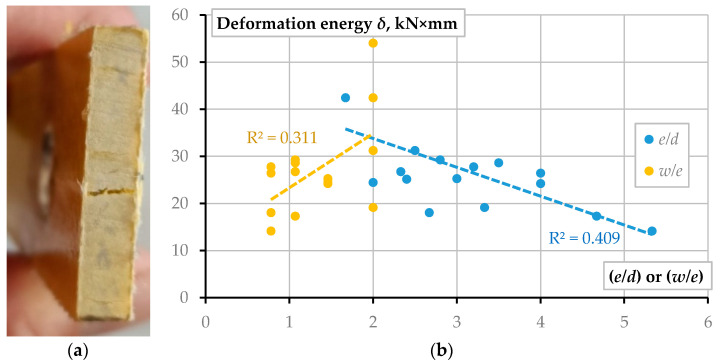
Summary of the test results: (**a**) A typical cleavage failure of the unidirectional pultruded core; (**b**) The deformation energy (Equation (1)) variation tendency.

Figure 6 demonstrates that the failure brittleness increases with the bolt diameter. Moreover, the failure suddenness, unfavorable in engineering applications, increases with decreasing the end distance (*e*). In this context, the bearing failure of the GFRP composite represents a less dangerous mode. This result agrees with the findings reported in the literature [4,6,35]. Additionally, in some instances, the bearing mode induces residual deformations similar to the peeling-out bolt failure when the joint can resist the load in the post-ultimate loading range. Table 5 summarizes the ultimate load-bearing capacity of all tested samples. In this table, columns “1” to “5” describe the responses of nominally identical specimens; **P***_u_* determines the averaged load-bearing capacity of five samples; **P***_th_* describes the theoretical resistance calculated from Equation (1), assuming the tensile strength of GFRP from Table 1; Δ is the relative difference between the theoretical and experimental resistance of the joints; and Equation (4) determined the efficiency ratio *η*. The following are significant conclusions raised from the results of Table 5:

- The theoretical model (Equation (1)) overestimates the ultimate resistance of all considered joints under the assumption of the GFRP strength estimated from the undrilled coupon tests (Table 1). This conclusion supports the previous finding related to the limited reinforcement efficiency in fibrous composites [18];
- The theoretical model (Equation (1)) demonstrates the best performance (estimated in terms of the prediction error Δ) for the relatively low *w*/*e* values (*w*/*e* ≤ 1). The condition *w*/*d* < 4 describes the most reliable prediction cases within this range, except for the B-12 specimens, where the cleavage failure was predominant (Table 4). This result supports the observations from the literature sources [4] and [12] but opposes the failure mechanisms identified in the article [11]. At the same time, this finding supports the insights related to the manufacturing technology’s effect on the mechanical performance of FRP components highlighted in Section 1;
- To the previous comment, the exceptionally low efficiency of the considered GFRP laminated composite (expressed in terms of the coefficient *η*) points out the necessity of additional means for strengthening the supposed drilled connections. The external laminate was unable to prevent the pultruded GFRP core failure. Figure 7a shows a typical cleavage failure of the unidirectional pultruded core under the outer sheet. References [12,13,16,35] describe several efficient strengthening techniques applicable for the considered plate connections.

As can be observed from the results in Table 3 and Table 4, cleavage damage typically occurs when *e*/*d* < 3, i.e., the combination of thin plate and large bolt diameter. Next, the cleavage damage transforms to shear-out damage with the plate thickness and end distance increase; still, this increase affects the damage stress, *σ_m_*, insignificantly. Finally, the shear-out damage transforms to the bearing damage with the condition *e*/*d* ≥ 4 is satisfied; at this stage, the stress *σ_m_* increase is significant. Therefore, the authors recommend this condition for the bolt connection design of the considered pultruded GFRP plates. Furthermore, the transverse tensile damage of the bolt connection does not occur when *w*/*d* ≥ 2.3. However, additional tests are necessary to verify the latter condition.

Table 6 shows the displacements corresponding to the ultimate load (Table 5) of all test specimens and averaged values of each specimen type, *u*_P_. This table also includes the corresponding averaged deformation energy, *δ_m_*, describing the joint failure ductility. The area below the ascending branch of the load-displacement diagram (e.g., Figure 6) describes this energy. In this study, the following linear approximation determines this parameter for simplification purposes:

(6) $$\delta_{m}=P_{u}\cdot u_{P}/2$$

The statistical analysis demonstrated that among the parameters listed in Table 3, only *e*/*d* and *w*/*e* ratios significantly affect the energy *δ_m_*, opposing the *w*/*d* parameter’s importance reported in the literature [10,12].

Figure 7b shows the averaged energy values (Table 6) scattered along the influence parameters. The trend lines define the effect tendencies; the determination coefficients, *R*^2^, describe the scatter part, which could be explained by the variation of the variable, i.e., either *e*/*d* or *w*/*e* ratio. In other words, these results mean that the variation of the *e*/*d* and *w*/*e* ratios can explain 40.9% and 31.1% of the deformation energy alteration observed in the experiments. An interested reader can find a more detailed explanation of the coefficient *R*^2^ interpretation procedure in reference [36].

**Figure 8:**
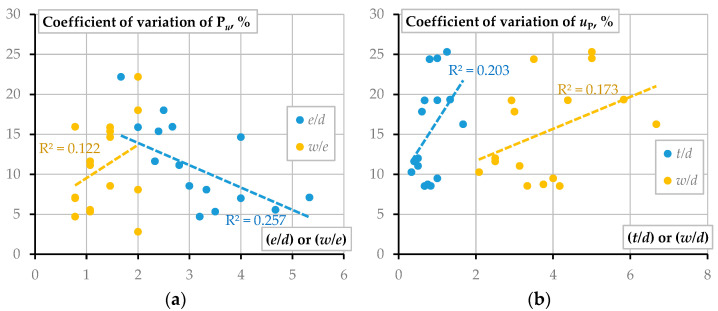
Variation of the test results: (**a**) Maximum load, **P***_u_*; (**b**) Deformation corresponding to **P***_u_*.

The multiple numbers of identical samples allow for assessing variation of the characteristic mechanical parameters of the bolted joints. Therefore, Table 5 and Table 6 include the variation coefficient values; Figure 8 summarizes the parametric analysis results of the scatter tendencies.

Figure 8a demonstrates the tendency of the variation coefficient of the ultimate load (Table 5), similar to the deformation energy trends shown in Figure 7b except for the linear approximation reliability—the models demonstrate the coefficient *R*^2^ equal to 0.257 for *e*/*d* ratio and 0.122 for *w*/*e* ratio. Figure 8b shows the opposite tendencies of the deformation corresponding to the ultimate load (Table 6)—only the *t*/*d* or *w*/*d* ratios have a detectable effect on the scatter. This trend is expectable and indicative of the scatter reduction with increasing the width and thickness of the plate. The remaining geometry parameters do not affect the spread of the characteristics presented in Table 5 and Table 6. In any case, however, the observed coefficients *R*^2^ are too low in developing a reliable prediction model.

## 5. Finite Element Model of the Bolted Connection

The theoretical analysis demonstrates systematic underestimation of the ultimate resistance expressed in the coefficient *η* terms (Equation (4))—none of the considered joints’ efficiency exceeds 20% (Table 5). Thus, this study employs the finite element (FE) analysis to identify possible ways to improve the bolted joint resistance in the pultruded GFRP plates.

### 5.1. The FE Model Description

The deformation problem is formulated in the 3D domain. A nonlinear FE analysis with Abaqus software predicts the load-bearing capacity of the bolted connection. The simulations include stress analysis, failure determination, and material stiffness degradation. The Abaqus software can determine the stress distribution, but the failure determination requires a damage criterion of the GFRP material. Therefore, this study employs the experimentally verified subroutine [33] describing the laminate failure, considering the pultruded GFRP as a single-layer composite plate. The GFRP plate is treated as transversely isotropic material with 17 solution-dependent state variables and five variables controlling finite element deletion, i.e., failure of the FE mesh structure. This subroutine uses the 3D Hashin [37] and Puck [38] failure criteria, describing fibers and polymer matrix damage processes. Table 7 describes the material model parameters of the GFRP plate; the default values determined the remaining constraints. The interested reader could find a detailed description of the material model in the reference [33].

The perfectly elastic material model describes the deformation behavior of steel. The 200 GPa modulus of elasticity and the 7820 kg/m^3^ density were assumed.

**Figure 9:**
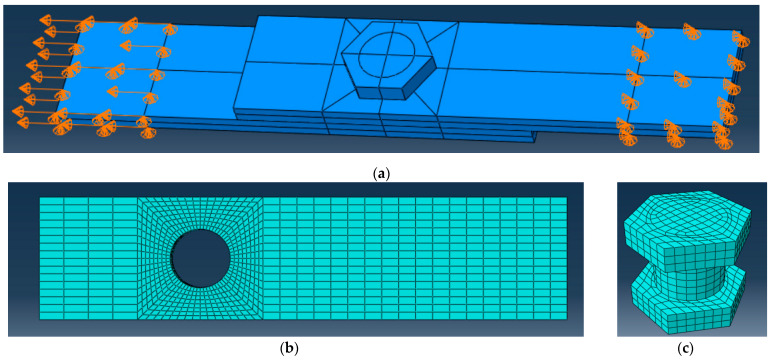
The FE model of the tested joints: (**a**) loading scheme and boundary conditions; (**b**) FE mesh of GFRP plate; (**c**) FE mesh of the steel bolt.

Figure 9 shows a FE model of the bolted joint, and Table 8 defines the model assumptions and FE size. Remarkably, the finite element size-sensitivity analysis was beyond the scope of this study, which uses the FE discretization as recommended in the references [19,21] to reduce the computation costs. This FE model requires three to four hours to reach the final convergence using the laptop with four parallel Intel Core I7-10750H CPU 2.60 processors and 16G RAM.

Figure 9a describes the loading and boundary conditions, which represent the physical tests (Section 2.2). Table 2 and Table 3 describe the specimen geometry. The iteratively applied displacement determines the loading situation. The loaded end deviation was prevented, fixing the plate in the “**2**” direction; the opposite end movements were fixed in the “**1**” and “**3**” directions.

Solid brick C3D8R, an eight-node linear brick, reduced the integration elements discretizing the FE model in Abaqus/Explicit solver; the relax stiffness hourglass-control method reduced zero-energy modes in the simulations. Figure 9b,c show the FE mesh. It can be observed (Figure 9b) that the square zone around the hole has refined mesh with six elements through the plate thickness. The steel bolt (Figure 9c) and nut are modeled as a single macroelement, neglecting the teeth on the contact surface with the GFRP plate.

### 5.2. Simulation Results

**Figure 10:**
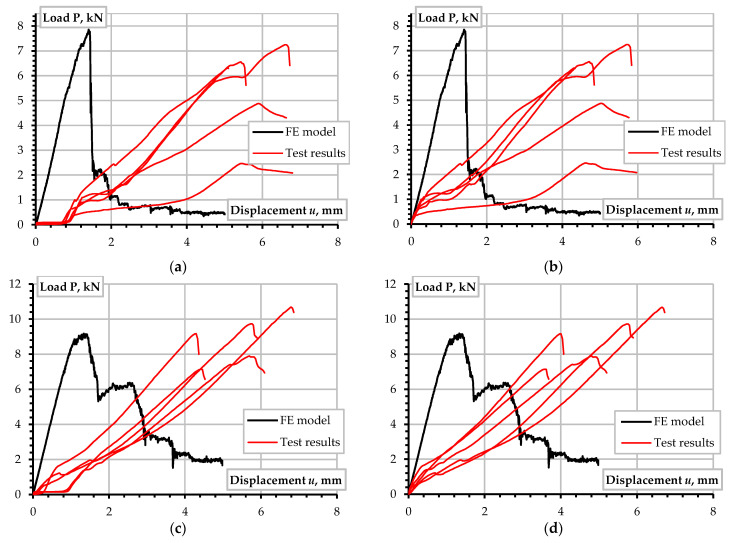
Load-displacement diagrams of the tested joints: (**a**,**b**) A-12 joint type before and after transformation of the experimental graphs; (**c**,**d**) The same diagrams of C-12 connections. Note: the diagrams of each graph show responses of five nominally identical test samples.

The simulation results of the A-12 and C-12 joints exemplify the numerical modeling. Figure 10 shows the deformation prediction results. Figure 10a,c, together with the simulated graphs, include the experimentally determined load-displacement diagrams. Several test specimens did not react to the tension load approximately until one mm displacement. This situation results from the insufficiently solid contact of the joint parts. Typically, several pre-loading cycles disappear such an effect [5]. However, the considered samples were not pre-loaded. Therefore, the authors theoretically shifted the experimental diagrams to the zero-point—Figure 10b,d demonstrate the modified graphs, which are the object for further analysis.

Let us consider the simulated diagrams the theoretical reference, describing the efficient behavior of the bolted connection. Thus, Figure 10b,d display that the initial slope of experimental and numerical graphs coincide, indicating the correctness of the assumed modulus of elasticity. However, the joins deform “elastically” until a relatively low load (approximately 1 kN). The experimental graphs’ deviation from the numerically predicted elastic line indicates the emergence of the defects of the bolted connection. These defects can result from the fiber cutting in the drilled hole, manual drilling flaws, and damaging the contact surface with the spiral teeth of the bolt. The decrease in the ultimate resistance of the joints could have similar origins [18,35]. At the same time, however, the test samples demonstrate a load-bearing capacity comparable to the predicted one.

In the considered examples, the numerical model predicts the 7.85 kN and 9.18 kN ultimate resistance of the A-12 and C-12 type joints that describe 25.7% and 2.9% error regarding the test results (6.25 kN and 8.92 kN, Table 5). In other words, the prediction error decreases with increasing the plate thickness and width, proving the reasonable adequacy of the developed simplified model, improving the theoretical predictions of Table 5. Moreover, the descending branch of the predicted load-displacement diagram (Figure 10) represents the brittle failure tendency observed in the tests.

**Figure 11:**
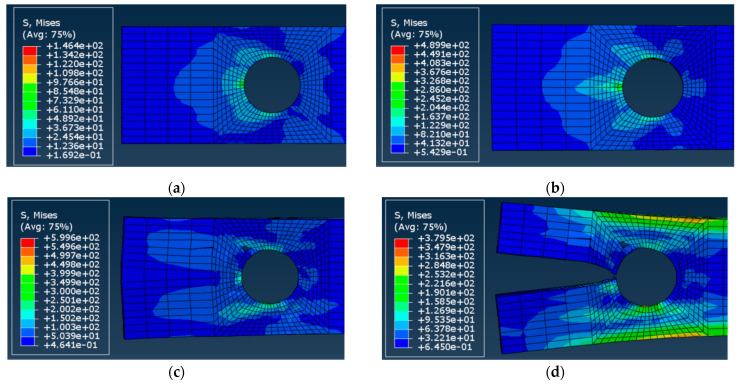
Numerically predicted deformation behavior and stress distribution in the A-12 joint (refer to Figure 10b for the deformation stages): (**a**) Displacement *u* = 0.259 mm, load **P** = 1.500 kN; (**b**) *u* = 1.040 mm, **P** = 6.500 kN; (**c**) *u* = 1.350 mm, **P** = 7.630 kN; (**d**) *u* = 2.440 mm, **P** = 0.798 kN.

Figure 11 illustrates the failure mechanisms of the A-12 joint hidden during the experimental loading. During the simulation process, the loaded surface of the hole was squeezed first. Then, the fiber and matrix were compressed and damaged; the matrix tensile damage occurred behand the compressed surface. Finally, the splitting damage occurred instantaneously, reaching the ultimate resistance of the GFRP material.

**Figure 12:**
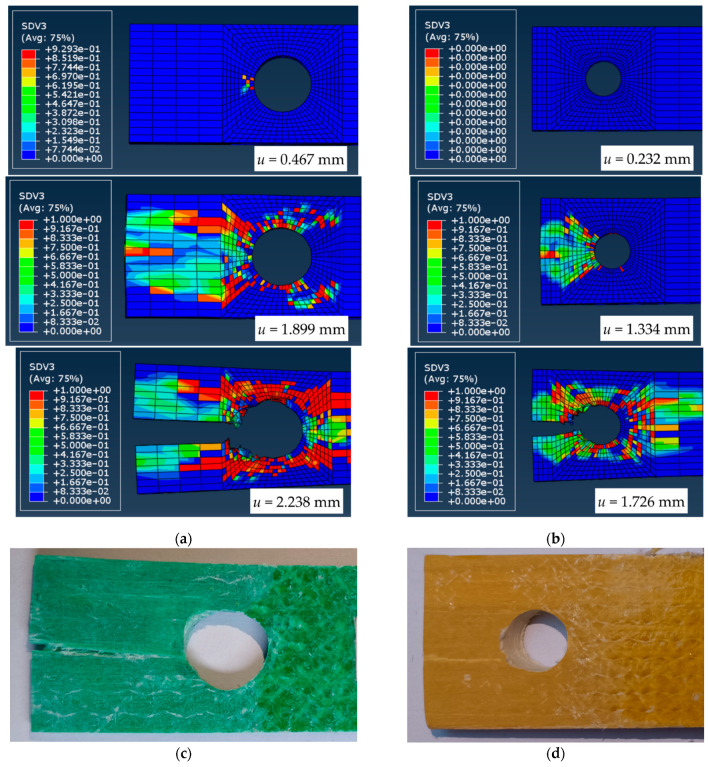
The simulated damage stages and test results comparison: (**a**,**b**) The numerically predicted damage stages of the A-12 and C-12 joints; (**c**,**d**) The corresponding failure patterns.

Figure 12 compares the deformation behavior of the A-12 and C-12 joints expressed in the damage factor SDV3 terms—the factor SDV3 = 0 corresponds to the undamaged material; SDV3 = 1 defines the complete failure, describing the limit when the subroutine eliminates the damaged finite element from the model. The simulation results’ consistency with the physical test outcomes proves the model’s adequacy. On the one hand, however, the authors point out the illustrative nature of Figure 11 and Figure 12. On the other hand, the developed numerical model can serve as an efficient reference for further developing mechanically fastened joints employing GFRP components.

## 6. Conclusions

This manuscript investigates the mechanical performance of a single-bolt connection of pultruded GFRP plates. The research variables were plate thickness and width, end distance, and hole diameter. The test program included four types of plate samples. Each testing group consisted of four joint geometries; five nominally identical joints were produced, resulting in 80 connections tested. The theoretical model reported in the literature determined the connection efficiency. Additionally, finite element (FE) Abaqus software simulated the bolted joints, employing the user-defined subroutine experimentally verified in the literature. The following conclusions result from this study:

- The test results demonstrate that the failure brittleness increases with the bolt diameter, *d*. In addition, the failure suddenness, unfavorable in engineering applications, increases with decreasing the end distance, *e*. In this context, the bearing failure of the GFRP composite represents a less dangerous mode, supporting the findings reported in the literature;
- Hart-Smith’s theoretical model overestimates the ultimate resistance of all considered joints under the assumption of the GFRP strength estimated from the undrilled coupon tests. The best agreement between the theoretical and experimental results corresponds to the relatively low width-to-end distance ratio (*w*/*e* ≤ 1). The width-to-bolt diameter ratio *w*/*d* < 4 describes the most reliable prediction cases within the above range. However, the exceptionally low efficiency of the considered GFRP laminated composite points out the necessity of additional means for strengthening the drilled connections;
- The statistical analysis demonstrated that only *e*/*d* and *w*/*e* ratios significantly affect the deformation energy of the bolted connections, opposing the *w*/*d* parameter importance reported in the literature. The dissipated energy amount demonstrated positive correlation with *w*/*e* ratio (*R*^2^ = 0.311) and negative relationship with *w*/*e* parameter (*R*^2^ = 0.409). In other words, these results mean that the variation of the *e*/*d* and *w*/*e* ratios can explain 40.9% and 31.1% of the deformation energy alteration observed in the experiments. The authors recommend the end distance-to-bolt diameter condition *e*/*d* ≥ 4 for the bolted connection design of the considered pultruded GFRP plates;
- The variation coefficient of the ultimate load revealed a similar tendency as above except for the linear approximation reliability—the regression models demonstrated the coefficient *R*^2^ equal to 0.257 for *e*/*d* ratio and 0.122 for *w*/*e* ratio. On the contrary, only the *t*/*d* or *w*/*d* ratios had a detectable effect on the scatter of the deformation corresponding to the ultimate load. However, the observed coefficients *R*^2^ are too low in developing a reliable prediction model;
- The theoretical analysis established systematic underestimation of the ultimate resistance—none of the considered joints’ efficiency exceeded 20%. Hence, this study employed the FE analysis to identify possible ways to improve the bolted joint resistance in the pultruded GFRP plates. The simulation results revealed that the joins deformed “elastically” until a relatively low load (not exceeding 1 kN). The experimental graphs’ deviation from the numerically predicted elastic line indicated the emergence of the defects of the bolted connection, which could result from the fiber cutting in the drilled hole, manual drilling flaws, and damaging the contact surface with the spiral teeth of the bolt. Thus, the strengthening procedures can improve the mechanical resistance of the joints, and the numerical model explicitly estimates the strengthening efficiency, quantifying the difference between the physical tests and predicted joint performances;
- In the considered examples, the numerical prediction error decreases from 25.7% to 2.9% with increasing the plate thickness (from 4 mm to 8 mm) and width (from 25 mm to 35 mm), proving the reasonable adequacy of the simplified model.

## Figures and Tables

**Table 1:** Mechanical parameters of the GFRP material.

Parameter	Value
Tensile strength (MPa)	475.3
Compression strength (MPa)	175.2
Modulus of elasticity of longitudinal tension (GPa)	33.3
Modulus of elasticity of longitudinal compression (GPa)	53.3
Modulus of elasticity of transverse tension (GPa)	3.68
Modulus of elasticity of transverse compression (GPa)	6.16
Poisson’s ratio (–)	0.27

**Table 2:** Geometry parameters of the different sample types (dimensions are in mm).

Parameter	Type A	Type B	Type C	Type D
Thickness, *t*	4	6	8	10
End distance, *e*	32	28	24	20
Width, *w*	25	30	35	40
Length, *l*	105	160	160	180

**Table 3:** Configuration of the test samples.

Type	Joint	*d* (mm)	*e*/*d*	*w*/*d*	*t*/*d*	*w*/*e*
A	A-6	6	5.33	4.17	0.67	0.78
	A-8	8	4.00	3.13	0.50	0.78
	A-10	10	3.20	2.50	0.40	0.78
	A-12	12	2.67	2.08	0.33	0.78
B	B-6	6	4.67	5.00	1.00	1.07
	B-8	8	3.50	3.75	0.75	1.07
	B-10	10	2.80	3.00	0.60	1.07
	B-12	12	2.33	2.50	0.50	1.07
C	C-6	6	4.00	5.83	1.33	1.46
	C-8	8	3.00	4.38	1.00	1.46
	C-10	10	2.40	3.50	0.80	1.46
	C-12	12	2.00	2.92	0.67	1.46
D	D-6	6	3.33	6.67	1.67	2.00
	D-8	8	2.50	5.00	1.25	2.00
	D-10	10	2.00	4.00	1.00	2.00
	D-12	12	1.67	3.33	0.83	2.00

**Table 4:** Configuration of the test samples.

Type	Group	*d* × *t* (mm^2^)	P*_u_* (kN)	*σ_m_* (MPa)	Failure Mode
A	A-6	24	5.14	214.2	B
	A-8	32	7.38	230.6	B
	A-10	40	7.67	191.8	C
	A-12	48	6.25	130.2	C
B	B-6	36	7.82	217.2	BF
	B-8	48	11.51	239.8	S
	B-10	60	11.58	193.0	S,C
	B-12	72	10.08	140.0	C
C	C-6	48	7.47	155.6	S
	C-8	64	9.32	145.6	S,C
	C-10	80	9.72	121.5	S,C
	C-12	96	8.92	92.9	S,C
D	D-6	60	6.85	114.2	S
	D-8	80	9.83	122.9	S
	D-10	100	14.95	149.5	S,D
	D-12	120	13.05	108.8	S,D

**Table 5:** The load-bearing capacity summary of all test specimens.

Joint	“1”	“2”	“3”	“4”	“5”	P*_u_* (kN)	P*_th_* (kN)	Δ (%)	*η* (%)
A-6	5.68	5.05	5.30	4.71	4.97	5.14 ± 7.1% ^‡^	7.79	51.4	11.4
A-8	7.32	8.20	7.47	7.10	6.82	7.38 ± 7.0%	8.91	20.7	16.4
A-10	7.96	7.50	7.52	7.23	8.12	7.67 ± 4.7%	9.34	21.9	17.0
A-12	2.47 *	4.88	6.31	7.25	6.56	6.25 ± 16.0%	9.16	46.6	13.8
B-6	7.44	8.26	8.30	7.71	7.40	7.82 ± 5.6%	13.09	67.3	9.1
B-8	10.75	11.50	12.27	11.94	11.09	11.51 ± 5.3%	15.52	34.8	13.5
B-10	12.62	11.15	13.19	10.04	10.91	11.58 ± 11.2%	16.99	46.7	13.5
B-12	8.36	10.99	10.42	9.44	11.18	10.08 ± 11.7%	17.57	74.3	11.8
C-6	5.61	7.70	7.52	8.33	8.21	7.47 ± 14.7%	17.80	138.2	5.6
C-8	8.03	10.20	9.34	9.65	9.37	9.32 ± 8.6%	21.47	130.4	7.0
C-10	7.97	8.54	11.13	11.30	9.65	9.72 ± 15.4%	24.07	147.7	7.3
C-12	7.14	10.68	7.89	9.16	9.73	8.92 ± 15.9%	25.64	187.4	6.7
D-6	6.51	6.12	7.50	7.24	6.90	6.85 ± 8.1%	22.31	225.6	3.6
D-8	9.38	12.84	8.20	9.74	9.01	9.83 ± 18.0%	27.16	176.2	5.2
D-10	14.62	15.45	14.48	14.86	15.31	14.95 ± 2.8%	30.83	106.3	7.9
D-12	13.09	15.49	16.28	10.82	9.55	13.05 ± 22.2%	33.41	156.0	6.9

* The average resistance did not account for this exceptional value. ^‡^ This number represents the mean ± coefficient of variation (in percent).

**Table 6:** The displacement corresponding to the ultimate load and deformation energy.

Joint	“1”	“2”	“3”	“4”	“5”	*u*_P_ (mm)	*δ_m_* (kN·mm)
A-6	5.20	5.04	6.26	5.49	5.47	5.49 ± 8.5% ^‡^	14.12 ± 11.3% ^‡^
A-8	7.45	6.77	8.38	6.36	6.78	7.15 ± 11.1%	26.40 ± 13.6%
A-10	8.00	7.94	6.17	6.49	7.49	7.22 ± 11.6%	27.74 ± 14.7%
A-12	5.44	5.88	5.08	6.60	5.44	5.69 ± 10.3%	18.03 ± 34.5%
B-6	3.69	2.99	4.69	5.65	5.18	4.44 ± 24.5%	17.29 ± 23.4%
B-8	4.68	5.00	5.26	5.46	4.38	4.96 ± 8.8%	28.61 ± 13.5%
B-10	5.63	4.96	4.84	3.65	5.98	5.01 ± 17.8%	29.22 ± 23.0%
B-12	4.70	5.76	4.87	4.83	6.09	5.25 ± 12.0%	26.71 ± 22.6%
C-6	4.46	6.25	7.88	6.69	6.59	6.37 ± 19.3%	24.22 ± 28.3%
C-8	4.28	6.91	5.59	5.44	4.56	5.36 ± 19.3%	25.23 ± 26.7%
C-10	3.68	4.47	6.44	6.23	4.33	5.03 ± 24.4%	25.13 ± 38.7%
C-12	4.44	6.79	5.68	4.28	5.76	5.39 ± 19.2%	24.44 ± 32.6%
D-6	5.85	4.75	5.14	7.01	5.06	5.56 ± 16.3%	19.13 ± 20.7%
D-8	4.65	7.93	5.20	5.42	7.92	6.23 ± 25.3%	31.22 ± 39.7%
D-10	6.36	7.60	7.84	7.72	6.62	7.23 ± 9.5%	54.02 ± 9.7%
D-12	6.69	6.85	6.67	5.48	6.56	6.45 ± 8.6	42.42 ± 27.4%

^‡^ This number represents the mean ± coefficient of variation (in percent).

**Table 7:** The GFRP material model parameters.

Parameter	Direction “1”	Direction “2”	Direction “3”
Modulus of elasticity, *E* (GPa)	33.27	3.68	3.68
Poisson’s ratio, *υ* (–)	0.27	0.27	0.40
Shear modulus, *G* (GPa)	1.6	1.6	1.0
Maximum tensile stress, *σ_t_* (MPa)	1800 *	31	31
Maximum compression stress, *σ_c_* (MPa)	450 *	50	50
Maximum shear stress, *τ* (MPa)	80	80	40
Density, ρ (kg/m^3^)	1600	1600	1600

* These values were tailored for adequate representation of the experimental load-bearing capacity.

**Table 8:** FE model assumptions.

Component	Model	Comment
Bolt and screw-nut	Perfectly elastic	A single macroelement with ~1.4 mm FE size
Bolt and GFRP plate contact	Hard contact	The bolt was fit without clearance; the teeth surface is neglected; the penetration is impossible
GFRP plate	Progressive damage model	Table 7 and reference [33] describe the model parameters with ~2.5 mm (plate ends) and ~1.2 mm (around the bolt hole) FE size
Contact between GFRP plates	Hard contact	The friction coefficient = 0.2; the penetration is impossible

## Data Availability

Not applicable.
